# Alpha-fetoprotein-producing early rectal carcinoma: a rare case report and review

**DOI:** 10.1186/s12957-015-0590-x

**Published:** 2015-05-12

**Authors:** Hiroyuki Anzai, Shinsuke Kazama, Tomomichi Kiyomatsu, Takeshi Nishikawa, Toshiaki Tanaka, Junichiro Tanaka, Keisuke Hata, Kazushige Kawai, Hironori Yamaguchi, Hiroaki Nozawa, Takamitsu Kanazawa, Tetsuo Ushiku, Soichiro Ishihara, Eiji Sunami, Masashi Fukayama, Toshiaki Watanabe

**Affiliations:** Division of Surgical Oncology, Department of Surgery, Faculty of Medicine, The University of Tokyo, 7-3-1 Hongo, Bunkyo-ku, Tokyo 113-8655 Japan; Department of Pathology, The University of Tokyo, 7-3-1 Hongo, Bunkyo-ku, Tokyo 113-8655 Japan

**Keywords:** Alpha-fetoprotein, Rectum, Early colon cancer, Adenocarcinoma

## Abstract

**Background:**

Alpha-fetoprotein (AFP)-producing rectal cancer is very rare, and this type of cancer frequently metastasizes to the liver with a poor prognosis. To date, only 11 cases of AFP-producing colorectal cancer have been reported.

**Case presentation:**

A 41-year-old woman was first presented to the hospital for anal bleeding. An elevated tumor with a central shallow depression in the lower rectum was detected by colonoscopy. Transanal excision was performed, and the histology revealed adenocarcinoma. Further immunohistopathological examination revealed that the tumor was an AFP-producing adenocarcinoma of the rectum. Although local resection was performed 2 months before the diagnosis of AFP tumor, the serum AFP level was normal. The depth of the submucosal invasion was 5,000 μm, and there was venous invasion. Also, no lymphatic invasion was detected. Therefore, additional surgical resection with lymph node dissection was conducted, and the patient underwent laparoscopic intersphincteric resection. No residual cancer was identified in the surgical specimens, and there was no evidence of lymph node metastasis. The patient was discharged 18 days postoperatively, and 12 months after the operation, there are no signs of recurrence.

**Conclusion:**

To the best of our knowledge, this is the first case of an AFP-producing rectal cancer that was diagnosed at an early stage.

## Background

Alpha-fetoprotein (AFP), a serum glycoprotein with a molecular weight of approximately 70 kDa, develops during gestation and is produced from fetal liver and yolk sac [1]. It was first described in 1963 by Abeleb *et al*. [2]. Immediately after birth, serum AFP levels are high, approximately 10,000 ng/mL but decrease rapidly, and by the second year of life and thereafter are less than 10 ng/mL. Some tumors produce AFP and lead to an increase in serum AFP levels. Therefore, AFP is a useful tumor marker in the diagnosis of tumors, such as hepatocellular carcinomas, hepatoblastoma, and yolk sac tumors [3-5]. AFP-producing tumors have mainly been reported in organs originating from the foregut endoderm [6]. The majority of AFP-producing cancers originate from the stomach, bile duct, and pancreas. However, AFP-producing colorectal cancer is extremely rare because the colorectum originates from the hindgut endoderm. Only 11 cases of AFP-producing colorectal cancer have been reported in English literature to date. Here, we report a case with early rectal cancer diagnosed as an AFP-producing tumor by immunohistochemistry. AFP-producing tumors have been reported to frequently metastasize to the liver and have a poor prognosis. However, the tumor in the present case was diagnosed at an early stage and no distant metastases were detected simultaneously [7-17]. To the best of our knowledge, this is the first case of an early diagnosis of an AFP-producing rectal cancer reported in English literature.

## Case presentation

**Figure 1:**
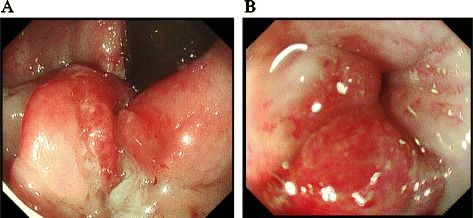
Colonoscopy findings. **(A)** Macroscopic evaluation by colonoscopy showed an elevated tumorous lesion in the lower rectum. A shallow depressed area can be seen at its center. **(B)** The surface of the elevated tumor showed redness.

**Figure 2:**
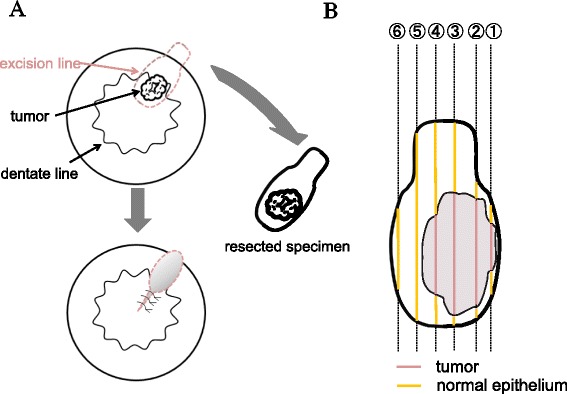
Schematic drawing of intraoperative situation. **(A)** Schematic drawing of intraoperative situation. **(B)** A schematic drawing of resected specimen.

**Figure 3:**
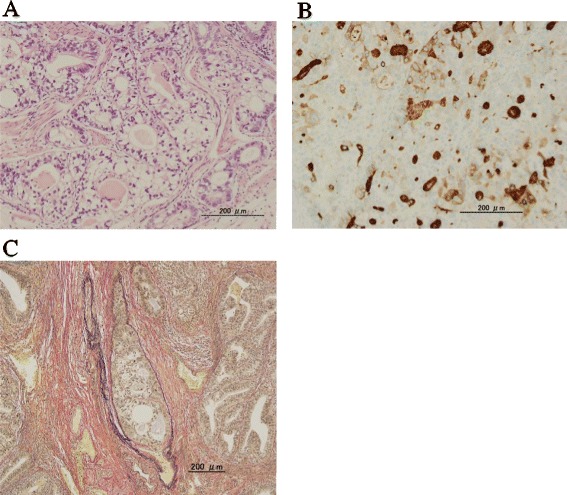
Microscopic findings. **(A)** Microscopic evaluation with hematoxylin-eosin staining of the tumor reveals columnar neoplastic cells with clear cytoplasm (original magnification, ×20). **(B)** Immunohistochemical staining of the tumor using an antibody against AFP. Diffused and strong positive staining is observed in the cytoplasm of the neoplastic cells (original magnification, ×20). **(C)** Elastic Van Gieson staining of the tumor reveals venous invasion by the adenocarcinoma (original magnification, ×25).

**Figure 4:**
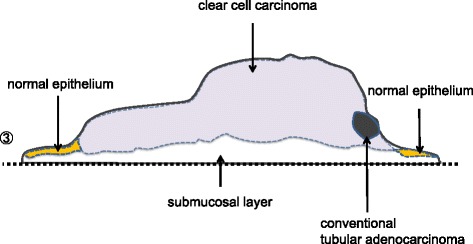
Histological mapping of cut surface ③.

A 41-year-old woman first noticed anal bleeding in December 2013. She had initially presented to a local hospital, and a colonoscopy was performed. Colonoscopy revealed an elevated tumor of approximately 15 mm in diameter with a central shallow depression in the lower rectum. It appeared similar to mucosal prolapse syndrome, and the histopathology of the biopsy specimen revealed no malignancy (Figure 1). Transanal excision of the elevated tumor was performed. The histopathological diagnosis of the tumor revealed a moderately differentiated adenocarcinoma. The patient was referred to our hospital for further investigation. Laboratory data investigations revealed normal serum hemoglobin. Tumor markers, such as carcinoembryonic antigen, carbohydrate antigen 19-9, and serum anti-p53 antibody, were within the normal ranges at 1.2 ng/ml (normal range 0 to 5.0 ng/ml), 11 U/ml (normal range 0 to 37.0 U/ml), and 0.40 U/ml (normal range 0 to 0.40 U/ml), respectively. Although elevated AFP levels take several months to normalize after the resection, the serum levels of 2 months after resection were within normal limits at 2 ng/ml (normal range 0 to 10.0 ng/ml). Abdominal computed tomography did not reveal liver metastasis, enlarged lymph nodes, or peritoneal metastasis, and the resected tumor was re-examined at our hospital. The schematic drawing of intraoperative situation is shown in Figure 2. Microscopically, the tumor comprised columnar neoplastic cells with clear cytoplasm showing tubular structure and focal solid growth by examination of the hematoxylin-eosin staining (Figure 3A). Most of the tumor was clear cell carcinoma, and the conventional adenocarcinoma present very focally (Figure 4). Immunohistochemistry using an anti-AFP antibody demonstrated diffused and strong positive staining of the cytoplasm of the neoplastic cells (Figure 3B). These results led to the diagnosis of AFP-producing adenocarcinoma of the rectum. The depth of the submucosal invasion was 5,000 μm, and there was a positive venous invasion (Figure 3C). Both these findings were suggestive of possible lymph node metastasis; therefore, we advocated additional surgical resection with lymph node dissection. Furthermore, the patient underwent laparoscopic intersphincteric resection in March 2014. The histopathological report revealed no residual tumor in the surgical specimens and no lymph node metastasis. The patient was discharged 18 days postoperatively, and 12 months later, there are no signs of recurrence.

## Conclusions

We report on a patient with AFP-producing rectal cancer diagnosed at an early stage. Although local resection was performed 2 months before the diagnosis of AFP tumor, the serum AFP levels of 2 months after the initial operation were normal. In Japanese literature, there are only two reported cases of AFP-producing colorectal disease detected in the early stages, which were T1 N0 and T2 N0 colorectal cancer, respectively[18,19]. AFP-producing cancer is defined by positively stained tumor of anti-AFP monoclonal antibody. Several reports describe that AFP-producing gastric cancer (AFP-GC) has an aggressive clinical course and poorer prognosis than AFP-negative GC. Interestingly, there are similarities with AFP-producing colorectal cancer and AFP-GC that it rapidly progresses and frequently metastasizes into the liver and show poor prognosis.

In present case, immunohistochemically evaluated glypican-3 expression, which is reported to be a sensitive marker for AFP-GC, was positive [20]. The immunohistochemical feature of present case was similar to AFP-GC. However, the histological characterization of AFP-producing colorectal cancer differs from AFP-GC. Although the most common subtype of AFP-GC is poorly differentiated carcinoma, poorly to moderately differentiated carcinoma is commonly observed among AFP-producing colorectal cancer. Additionally AFP-producing colon cancer is extremely rare, with only 11 reported cases in English literature [7-17]. The clinicopathological findings of these cases are summarized in Table 1. Of these 11 cases, 10 had elevated serum levels of AFP. The rectum was the most common tumor site and almost all cases had extensive simultaneous liver metastasis. Of the 11 cases, 6 cases died of the primary AFP-producing tumor and 2 cases died of postoperative complications. In addition, although almost all reports demonstrate the poor prognosis of AFP-producing tumors, there was no distant metastasis or recurrence in the present case. Although there are several reports of an AFP-GC with features of hepatic differentiation, the mechanisms of AFP-producing rectal cancer or the hepatic differentiation remain obscure.

**Table 1:** **Clinical features of reported cases of AFP-producing colorectal carcinomas**

**Case**	**Author**	**Age/gender**	**Location**	**Pretherapy AFP level (ng/ml)**	**Macroscopic classification**	**Depth of invasion**	**Lymph node metastasis**	**Pretherapy Metastases**	**Histology**	**Prognosis**
1	Nakajima *et al*. [7]	50/M	R	3,018	NS	Prostate	+	Liver/lung	Mod-por	5M dead
2	Yu *et al*. [8]	54/M	R	5,126	Ulcerated	Serosal	+	Liver	Well	0M dead
3	Sato *et al*. [9]	43/M	R	7,060	Ulcerated	Extraserosal	+	Liver	Well-mod	4M dead
4	Hocking *et al*. [10]	39/F	S/C	7,200	NS	Perforation	+	Liver	^a^	1M dead
5	Kato *et al*. [11]	75/M	C	3,070	Ulcerated	NS	+	No	Por	4M dead
6	Taguchi *et al*. [12]	71/M	R	220,000	Ulcerated	Muscular	-	No	^b^	12M dead
7	Kurihara *et al*. [13]	67/M	T/C	10,978	Ulcerated	Serosal	+	Liver	Por	NS
8	Ishikura *et al*. [14]	48/F	S/C	6,600	Ulcerated	Subserosal	NS	Liver	Well	4M dead
9	Lattes *et al*. [15]	41/M	R	NS	Ulcerated	NS	+	Liver	Well-muc-sig	12M alive
10	Yachida *et al*. [16]	59/M	T/C	12,873	Ulcerated	Serosal	-	Liver	Well	2M dead
11	Fu *et al*. [17]	71/M	T/C	318	Ulcerated	Subserosal	-	No	Por	5Y alive
12	Present case	41/F	R	2	Elevated	Submucosal	-	No	Mod-por	2M alive

AFP = alpha-fetoprotein; NS = not stated; C = cecum; S/C = sigmoid colon; T/C = transverse colon; R = rectum; well = well differentiated adenocarcinoma; mod = moderately differentiated adenocarcinoma; por = poorly differentiated adenocarcinoma; muc = mucinous adenocarcinoma; sig = signet-cell-carcinoma; ^a^Adenocarcinoma showing hepatoid morphology; ^b^glandular differentiation consisted of columnar cancerous cells.

Several studies have demonstrated the histopathological factors that predict lymph node metastasis of T1 stage colorectal cancer. The rate of lymph node metastasis of submucosal cancer has been reported to be 6% to 12% [21]. The risk factors for lymph node metastasis in submucosal colorectal cancers include poor differentiation, lymphatic invasion, vascular invasion, deep submucosal invasion, or a positive resection margin [22-24]. In the present case, the risk factors of massive invasion and vascular invasion were detected, which led us to perform the additional surgery.

In conclusion, we report on an AFP-producing colon cancer diagnosed at an early stage, whereby early detection enabled a complete resection of the carcinoma. It is important to note that management of a patient with an elevated tumor with a shallow depression, even when no malignancy is detected, should include local excision for immunohistopathological analysis. Although serum AFP levels have been high in all previously reported cases, it is important to remember that serum AFP levels may not rise in the early stages of AFP-producing cancer. Further accumulation of data and investigation of AFP-producing colon cancer is necessary.

## Consent

Written informed consent was obtained from the patient for publication of this case report and accompanying images.
